# Alternation of inverse problem approach and deep learning for lens-free microscopy image reconstruction

**DOI:** 10.1038/s41598-020-76411-9

**Published:** 2020-11-19

**Authors:** L. Hervé, D. C. A. Kraemer, O. Cioni, O. Mandula, M. Menneteau, S. Morales, C. Allier

**Affiliations:** Univ. Grenoble Alpes, CEA, LETI, DTBS, 38000 Grenoble, France

**Keywords:** Microscopy, Interference microscopy, Cellular imaging

## Abstract

A lens-free microscope is a simple imaging device performing in-line holographic measurements. In the absence of focusing optics, a reconstruction algorithm is used to retrieve the sample image by solving the inverse problem. This is usually performed by optimization algorithms relying on gradient computation. However the presence of local minima leads to unsatisfactory convergence when phase wrapping errors occur. This is particularly the case in large optical thickness samples, for example cells in suspension and cells undergoing mitosis. To date, the occurrence of phase wrapping errors in the holographic reconstruction limits the application of lens-free microscopy in live cell imaging. To overcome this issue, we propose a novel approach in which the reconstruction alternates between two approaches, an inverse problem optimization and deep learning. The computation starts with a first reconstruction guess of the cell sample image. The result is then fed into a neural network, which is trained to correct phase wrapping errors. The neural network prediction is next used as the initialization of a second and last reconstruction step, which corrects to a certain extent the neural network prediction errors. We demonstrate the applicability of this approach in solving the phase wrapping problem occurring with cells in suspension at large densities. This is a challenging sample that typically cannot be reconstructed without phase wrapping errors, when using inverse problem optimization alone.

## Introduction

A lens-free microscope is a minimalist setup for in-line holography^1–3^. The lens-free records only intensity measurements in the sensor plane, the image of the sample is obtained through computation. A simple procedure consists in back-propagating the measurements, but in the absence of phase information, the reconstructed image is degraded by so-called ‘twin-image’ artefacts^4^. To overcome the lack of information, various inverse problem approaches have been used to better reconstruct the sample image, i.e. forward model based algorithms, which perform either parameter fitting and/or regularization with a gradient descent scheme^5–8^. However, if the optical thickness of the sample exceeds $\lambda /2$, the reconstructed image is still largely incorrect, as the phase values are reconstructed modulo $2\pi$ whereas true values can exceed this range. This is the case for cells in suspension and cells undergoing mitosis. Their images are systematically reconstructed with phase wrapping errors. This problem considerably limits the application of lens-free microscopy in live cell imaging. At low cell concentrations, it is possible to apply a simple positive phase constraint to correct the phase wrapping errors^9^. But at large cell concentrations, the accumulation of phase wrappings errors in the reconstruction prevents the use of this method. Recent publications have introduced the use of convolutional neural network (CNN) to address phase unwrapping in digital holography microscopy^10–15^. The results obtained by Zhao et al.^13^ demonstrate that the CNN solution is robust to unwrap phase containing heavy noise and aliasing. A successfull unwrapping phase images of living mouse osteoblast up to phase shift of $13\pi$ has been shown. In the work of Ren et al.^15^, the CNN solution applied to digital holography allows to reconstruct phase directly from the hologram. Different deep learning approaches have been successful in improving lens-free holographic reconstruction results^16,17^ but they do not address phase wrapping issues. A specific CNN could be trained to transform a lens-free reconstructed image into an image free of phase wrapping, as obtained with a quantitative phase imaging technique^18–20^. A similar approach have been used to transform images between different imaging techniques^21,22^. However, the performance of image reconstruction using CNN is compromised by the problems of hallucination, generalization and adversarial fragility^23^. The hallucination problem deals with insufficient network training, resulting in systematic CNN prediction errors. The generalization problem is a result of overtraining, where the CNN fails to process unseen data. At last, in the adversarial fragility problem the CNN can produce widely differing results after adding imperceptible variations to the input. In sum, deep-learning is a data-driven approach which can deliver an outcome very close to the reality, however with an intrinsic lack of confidence in the results owing to the above-mentioned problems.

**Figure 1:**
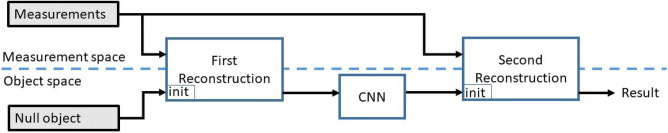
Overview of the data processing performed in the alternation approach.

In order to perform phase unwrapping in lens-free microscopy with a CNN based-solution and address issues of deep learning, here we propose an approach that alternates between the deep learning and inverse problem approach (see Fig. 1). We start the process with a first incorrect reconstruction of the cell sample image. The result is then fed into a CNN, which is improving the reconstruction by removing phase wrapping errors. The CNN prediction is next used as the initialisation of a second reconstruction. The latter returns the final result. In the best case, the alternation approach can lead to an accurate result found trough the CNN prediction which better initializes the inverse problem approach. If the CNN returns an inaccurate prediction with no concordance to the measurements, we expect that the second reconstruction algorithm will retrieve a solution with a better data fit. Note that this approach differs from the three methods based on deep learning recently reviewed in^24^. Our alternation approach is not an end-to-end deep learning engine, it does not predict the sample image from the raw acquisition. It is neither a single-pass physics-informed deep learning engine, applied only once after inversion of the raw acquisition^16,24^. Nor is it a physics-informed deep learning engine, running the regularization in place of a regular gradient-descent scheme^25,26^.

In this paper, we demonstrate the applicability of the proposed alternation approach in solving the phase wrapping problem that occurs in lens-free holographic reconstruction. The approach has been specifically developed for the reconstruction of images of cells in suspension. It has first been developed and assessed on simulations and next validated on experimental acquisitions.

## Results

### Validation on simulated datasets

**Figure 2:**
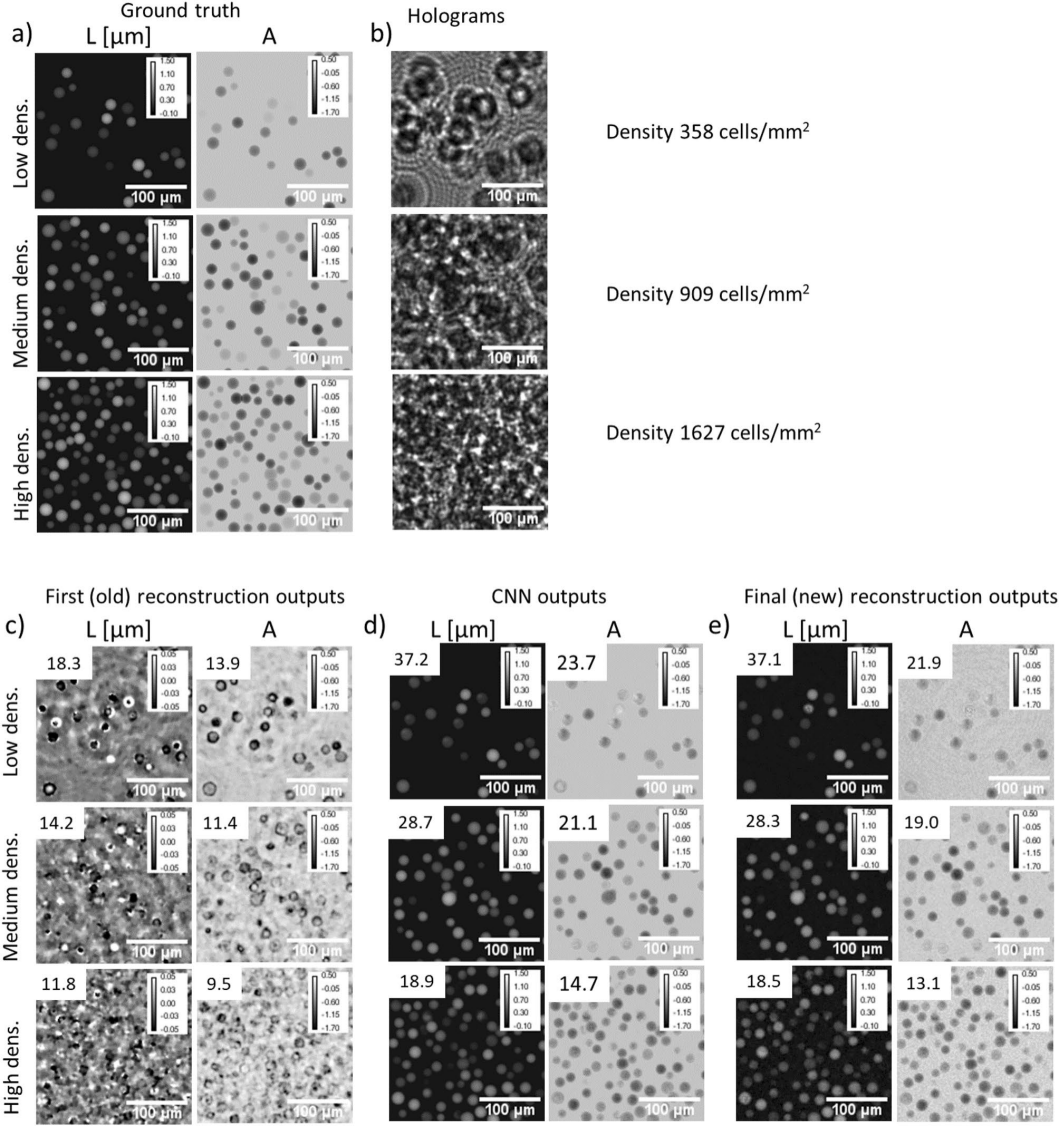
Results of the alternation reconstruction method obtained on the synthetic validation set at low density (358 cells/$\text{mm}^2$), medium density (909 cells/$\text{mm}^2$) and high density (1627 cells/$\text{mm}^2$). Subfigure (**a**) presents ground truth images (*L* and *A*) of the object, subfigure (**b**) presents holograms obtained by using Eq. (2). Results of first (old) holographic reconstruction (**c**), of the CNN step (**d**), and of the final (new) reconstruction (**e**). Images are $150\times 150$ pixels crops of the original images ($1.67\,\upmu\text{m}$ pitch). In (**c**,**d**,**e**), peak signal-to-noise ratio (PSNR) measurements are indicated in the top left of the results to assess the reconstruction versus ground truth.

**Figure 3:**
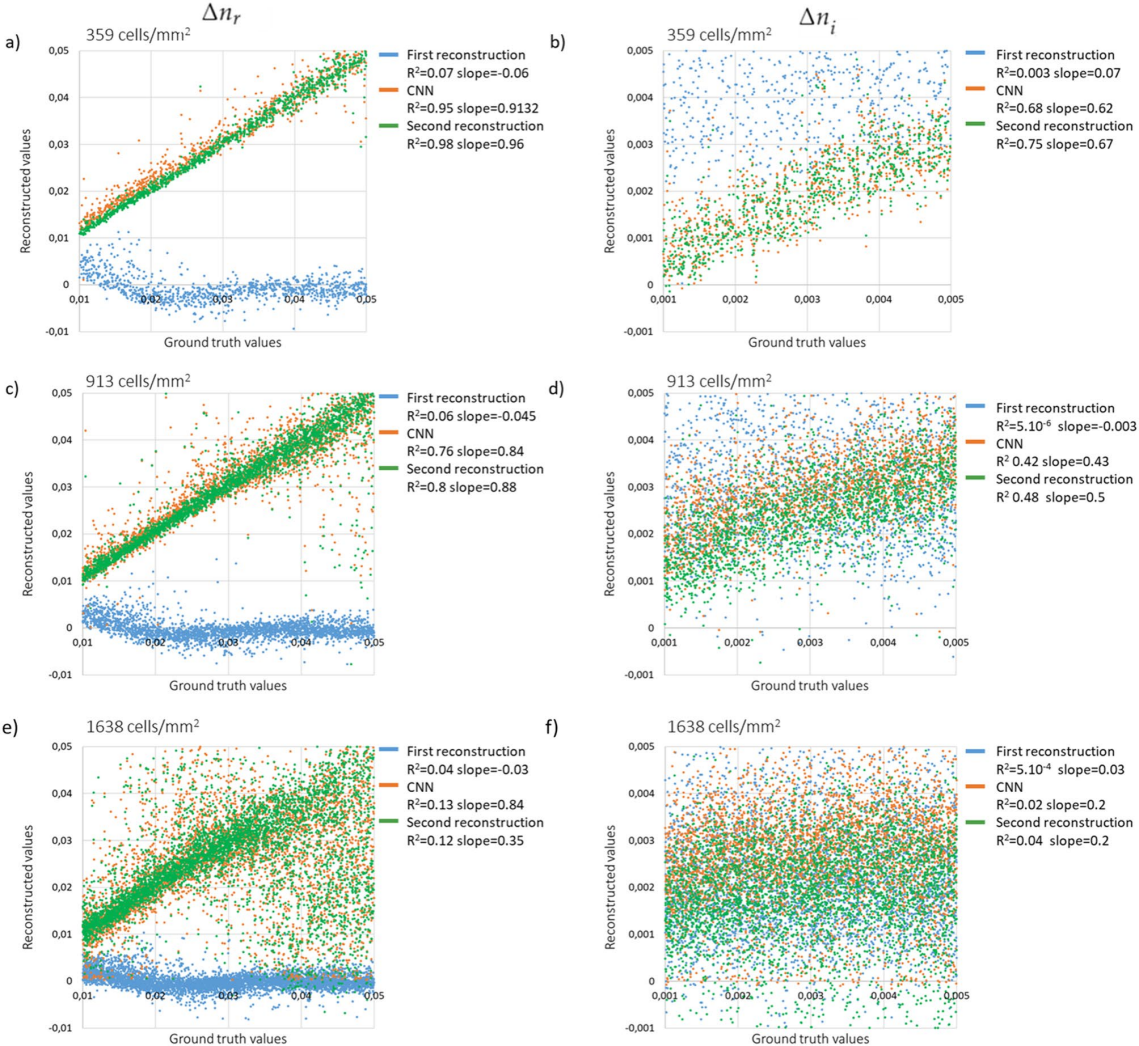
Reconstructed $\Delta n_{r\_recons}$ and $\Delta n_{i\_recons}$ as a function of the ground truth values. The three lines of the figure correspond to three different cell densities, namely 358, 909 and 1627 cells/$\text{mm}^2$ (see corresponding images in Fig. 2). The results obtained after each individual step of the alternation approach are shown, namely the first reconstruction (blue dots), the CNN output (orange dots) and the final reconstruction (green dots). The results of the linear regressions are indicated with values of slope and coefficient of determination ($R^2$).

Figure 2 shows the outcome of the proposed three-step reconstruction algorithm for three examples of the synthetic validation set corresponding to different cell densities—low, medium and high. The number of cells contained within the entire $1000\times 1000$-pixel image were 1001, 2547 and 4569 corresponding respectively to densities of 358, 909 and 1627 cells/$\text{mm}^2$. Note, the hologram of the high density case is speckle-like. As expected, objects recovered by the first (old) reconstruction, shown in Fig. 2c are degraded, with the presence of numerous wrapping errors in the OPD map (occurring when *L* exceeds $\lambda /2$). Absorption maps *A* show cells outlined with absorbing circle artefacts. In the high density case, the first holographic reconstruction results are barely intelligible. In comparison, *L* and *A* images predicted by the CNN (Fig. 2d) are better matched to the ground-truth images. The final (new) reconstruction results (Fig. 2e) have similar appearance as the CNN results. Figure S1 shows the convergence plots for these reconstructions, namely the data fidelity criterion, the regularization criterion and their sum as a function of the iterations (see Eqs. 3, 4 and 5). Notably, Fig. S1 shows that the CNN used for phase unwrapping systematically introduces a large deviation between the prediction of the model and the measurements. This error is corrected by the third step, which improves data matching and allows for a low regularization term. The peak signal-to-noise ratio (PSNR) values measured between results and ground truth are given in Fig. 2. The CNN increases the PSNR values by almost a factor two in comparison with the first reconstruction. The PSNR measured on the final reconstruction are slightly lower than what obtained on the CNN prediction. In order to better assess these results, Fig.  3 shows a quantitative comparison between reconstructed and ground truth values for the three different cell densities presented above. The values of the real and imaginary part of the cell refractive index, $\Delta n_{r\_recons}$ and $\Delta n_{i\_recons}$ respectively, were obtained from the reconstructions (see Eq. 7) and compared to their ground truth values. For the first reconstruction, even at low concentrations, there is no correlation between reconstructed and ground truth values. In comparison, the results obtained with the CNN and the second reconstruction are linearly correlated with the ground truth values. For the $\Delta n_{r\_recons}$ values, we found good correlations up to index values of 0.05 and up to a cell density of 900 cells/$\text{mm}^2$ (Fig.  3a,c). The slopes of the linear regressions are in the range of 0.84 to 0.96 and the coefficients of determination $R^2$ are all larger than 0.75. The best results are obtained with the second reconstruction which is slightly better than the CNN output. At the highest tested cell density of 1618 cells/$\text{mm}^2$ (Fig.  3e), values are correlated up to index values of 0.03. Regarding $\Delta n_{i\_recons}$, the reconstructed imaginary part of the cell refractive index, the results correlate with the ground truth values only at the low concentration of 360 cells/$\text{mm}^2$ (Fig.  3b). The slopes of the linear regressions are about 0.6 and the coefficients of determination $R^2$ about 0.7. Again the results obtained with the second reconstruction are slightly better than what was obtained with the CNN.

### Validation on experimental data

**Figure 4:**
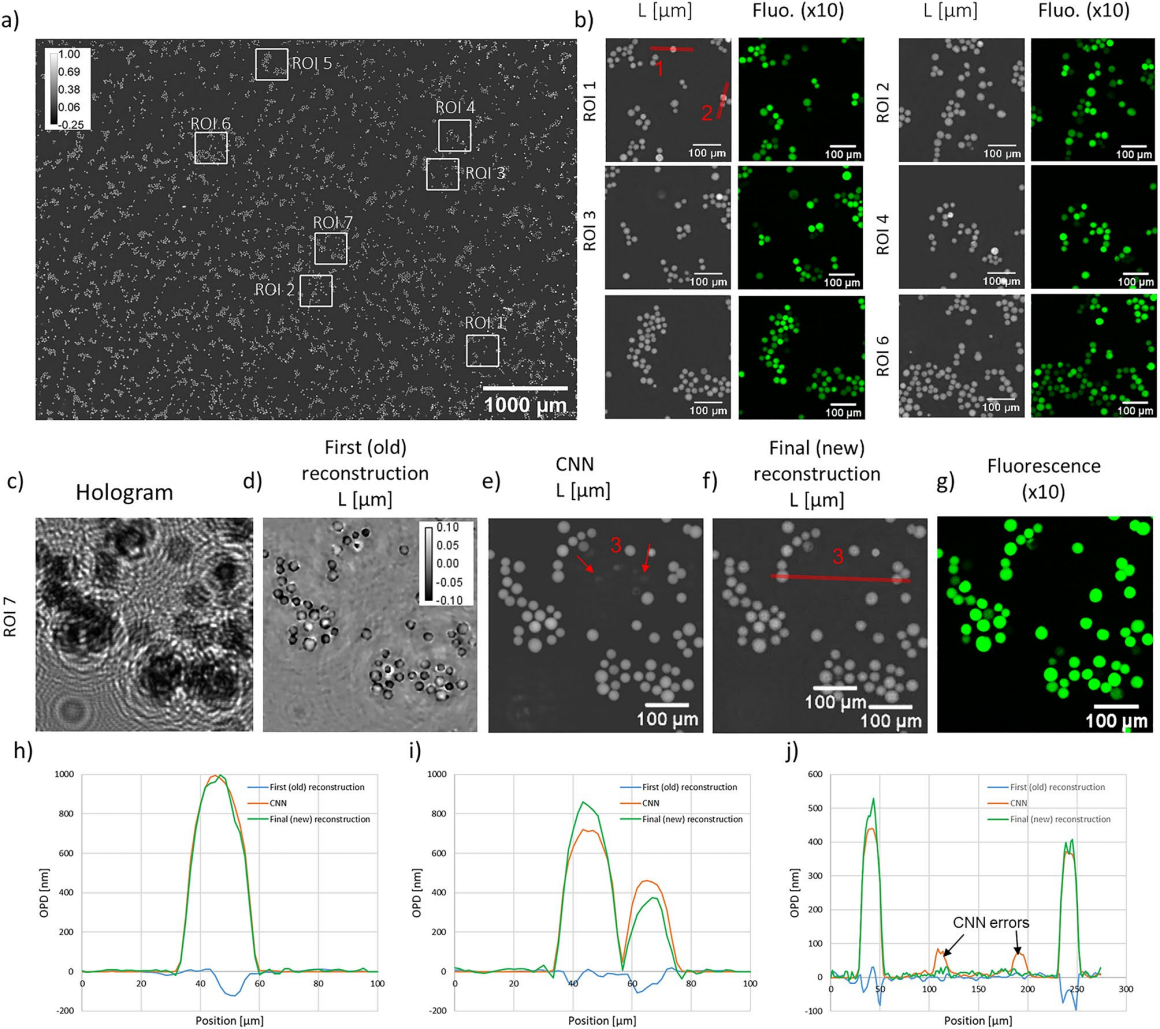
Application of the alternation reconstruction method on PC3 non-adherent cells at low density. Estimated number of cells $\approx 8450$, corresponding to a volumetric density of $\approx 14 \times 10^6$ cells/ml (measured in a $20\,\upmu\text{m}$ thick chamber) or a surface density of $\approx 290$ cells/$\text{mm}^2$. (**a**) Full field of view, final reconstruction. (**b**) Reconstructions of 6 selected regions of interest from image (**a**) and comparisons with their fluorescence microscope acquisitions. (**c**–**g**) Reconstruction of the seventh region of interest, with corresponding raw acquisition (**c**), first (old) reconstruction result (**d**), CNN output result (**e**), final (new) reconstruction result (**f**) and the comparison with the fluorescence acquisition (**g**). (**h**) OPD profiles through one cell (red line 1 in (**b**)). The maximum OPD on the final reconstruction is about 1000 nm which corresponds to a phase shift of about 5$\pi$. (**i**) OPD profiles through two cells (red line 2 in (**b**)). (**j**) OPD profile through two cells (red line in (**f**)). The black arrows indicate CNN errors (orange curve in (**j**) and red arrows in (**e**)) that are corrected by the last reconstruction (green curve).

**Figure 5:**
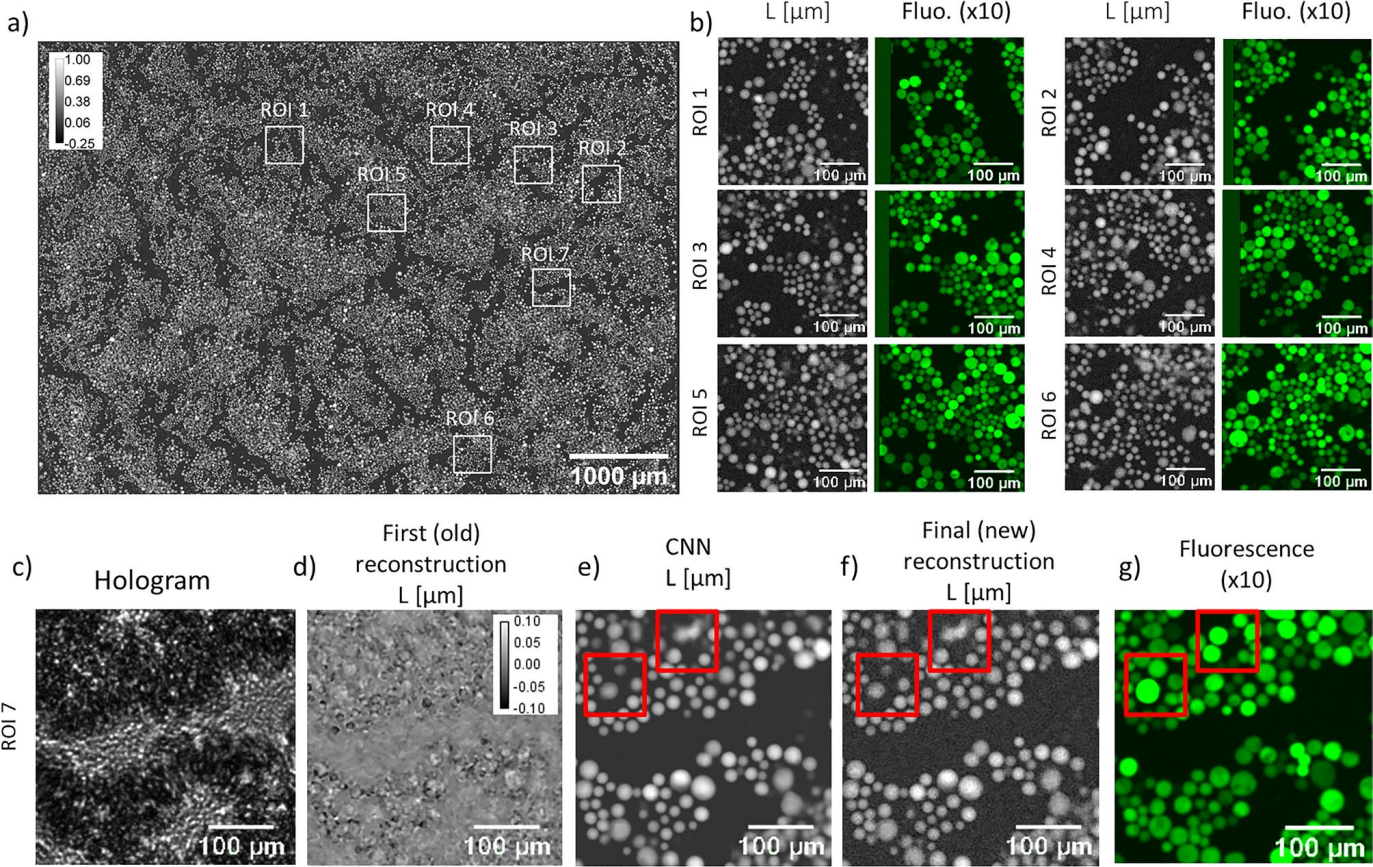
Application of the alternation reconstruction method on PC3 non-adherent cells at high density. Estimated number of cells: $\approx 34{,}000$ corresponding to a volumetric density of $\approx 57 \times 10^6$ cells/ml (measured in a $20\,\upmu\text{m}$ thick chamber) or a surface density of $\approx 1140$ cells/$\text{mm}^2$. (**a**) Full field of view final reconstruction. (**b**) Reconstructions of 6 selected regions of interest (**a**) and their comparisons with the fluorescence microscope acquisitions. (**c**–**g**) Reconstruction of the seventh region of interest, with corresponding raw acquisition (**c**), first (old) reconstruction result (**d**), CNN result (**e**), final (new) reconstruction result (**f**) and the comparison with the fluorescence acquisition (**g**). Red boxes in (**e**–**g**) highlight the discrepancies between the two modalities.

The alternation reconstruction method was further tested on the lens-free acquisition of PC3 cells in suspension. Figures 4 and 5 show the results obtained on PC3 cells measured in a 20 $\upmu\text{m}$ thick chamber at low density ($N=8450$ cells corresponding to a density of $14 \times 10^6$ cells/ml in Fig. 5) and high density ($N=34{,}000$ cells corresponding to a density of $57 \times 10^6$ cells/ml in Fig. 4). Distance *Z* was determined to be $1830\,\upmu\text{m}$, a network was trained for this distance. In the absence of reference measurements obtained with quantitative phase imaging techniques^18,19^, we can only discuss the reconstructed images in a qualitative way. Figures 4 and  5 show that the results obtained on experimental data are in line with the results obtained previously on the synthetic data (Fig. 2). The first (old) holographic reconstruction results are barely intelligible. In comparison, *L* and *A* images predicted by the CNN and obtained by the final (new) reconstruction present clear pictures of rounded cells. All phase wrapping problems are thus efficiently solved. The maximum obtained OPD values are about $1\,\upmu\text{m}$ which corresponds to a phase shift of approximately $5\pi$ (see Fig. 4h, $\lambda =405 \,\text{nm}$). In agreement with the simulated data, the last reconstruction step improves the matching between our model and measured data (see convergence plot in Fig. S1). This improvement brought by the third step can also be seen on the resulting image. Figure 4j points out errors introduced by the CNN but corrected by the third step. Fluorescence microscopy was used to validate the positions and the shapes of the cells in the reconstructed images. Based on the comparison between the two modalities, we conclude that reconstructions from our alternation approach recover well the low density sample and give satisfactory results at high densities. The red boxes in Fig. 5 show typical discrepancies which appear at large cell density in the presence of the largest cells. This in agreement with the simulation which pointed out that the CNN is effective at large cell density only up to a given refractive index value (Fig. 3c,d). With these results, we can therefore conclude that the alternation approach is effective up to a concentration of $\sim 1000$ cells/$\text{mm}^2$, in agreement with results of the simulation study.

**Figure 6:**
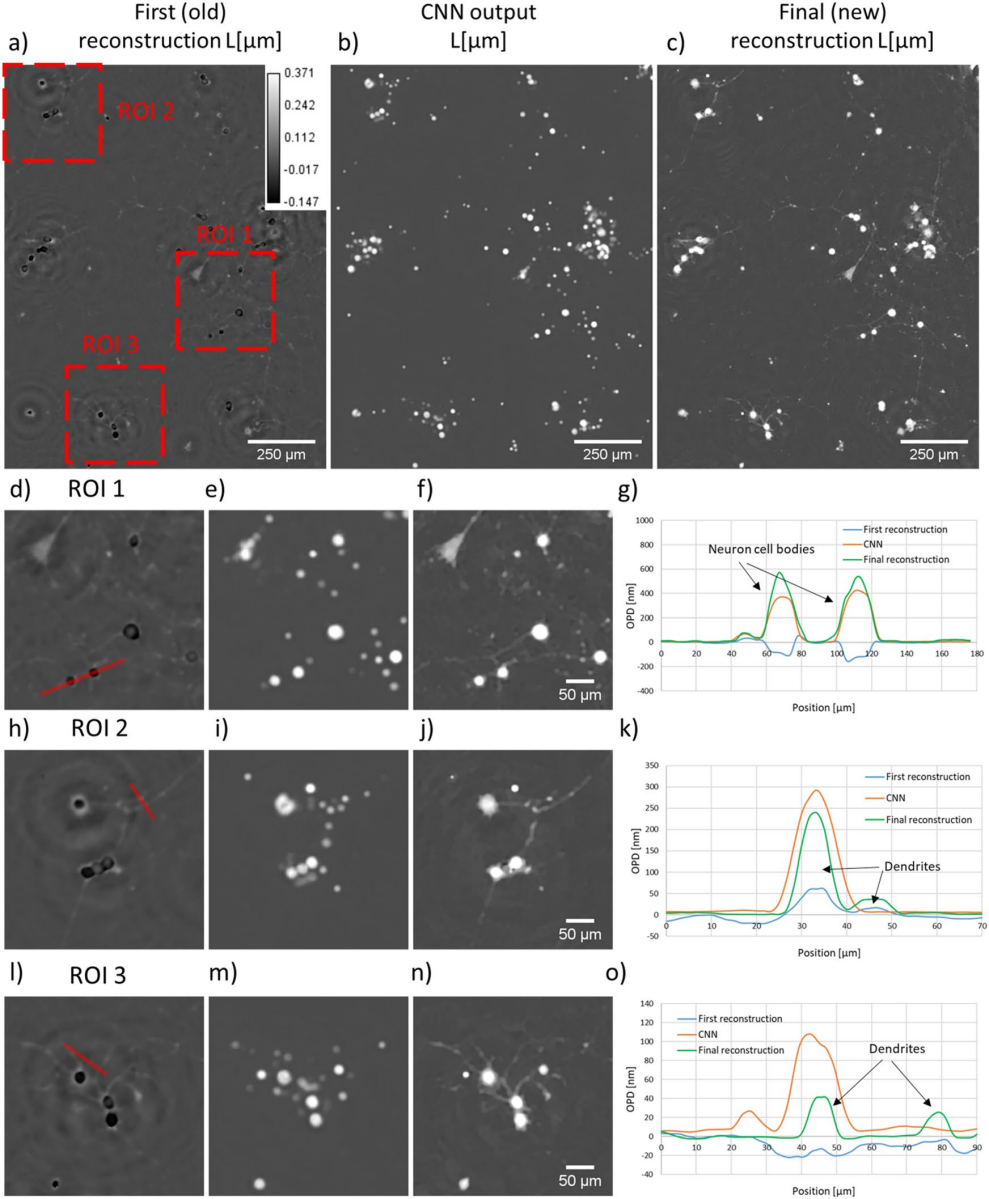
Results of the alternation reconstruction method applied to adherent PC12 cells treated with neuron growth factor. The first reconstruction results is shown in (**a**), CNN result in (**b**) and final reconstruction result in (**c**). (**d**–**o**) present detailed results corresponding to three regions of interest depicted by red boxes in (**a**). (**g**) OPD profile through two neuron cell bodies (red line in (**d**)). (**k**) OPD profile through two dendrites (red line in (**h**)). (**o**) OPD profile measured at the proximity of a cell, through two dendrites (red line in (**l**)).

As a generalization test, we applied the proposed alternation reconstruction approach to the acquisitions of different cell lines in suspension. Supplementary figures depict the reconstruction of cell culture in suspension of Molt4 cells (Fig. S2), Jurkat cells (Fig. S3) and CHO cells (Fig. S4). The reconstruction results are similar to that obtained on PC3 cells (Figs. 4 and 5). The CNN performs phase unwrapping while the last reconstruction step improves the matching between the model prediction and the measurement (see Fig. S1). As a second test, we applied the method to the reconstruction of a sample not considered during the CNN training, namely the acquisitions of an adherent mammalian cell line. Figure 6 shows the results obtained on the acquisition of adherent PC12 cells treated with neuron growth factor (NGF). Distance *Z* for this experiment was evaluated at $3500\,\upmu\text{m}$, a dedicated CNN was trained at this distance. Interestingly, in this case it is possible to clearly distinguish the three different steps of the alternation approach and their impact on the image reconstruction. After the first holographic reconstruction, the image appears degraded (Fig. 6a,d,h,l). The large thickness of the cells results in several phase wrapping artefacts. The neuron heads are reconstructed with negative values (see blue profile in Fig. 6g). Finer morphological features are not well reconstructed, for instance the neurites which are only 1 to 3 μm wide. The CNN prediction performs well phase unwrapping at the location of the neuron cell body (see orange profile in Fig. 6g), but the complexity of the scene is lost. The CNN prediction presents only rounded spots with positive phase values (Fig. 6b,e,i,m). Details such as the neurites are lost in the process (see orange profile in Fig. 6k). Obviously these details were not present in the training dataset, the CNN output is thus facing here a generalization problem. In addition, the CNN predicts the presence of rounded spots that are not present in the scene (see orange profile in Fig. 6o). The CNN output is thus facing here a hallucination problem. Notably, the final reconstruction corrects these CNN predictions (see green profiles in Fig. 6k,o). This example shows the good complementarity of the two algorithms. The CNN addresses the phase unwrapping and the final reconstruction retrieves the finer details that are lost in the CNN prediction. The PC12 cells could therefore be well reconstructed using the alternation approach whereas the complexity of these images was not accounted in the CNN training data set. As a last example, Fig. S5 depicts a case, namely the culture of adherent fibroblast cells, where the three steps approach was not successful. We observe errors introduced by the CNN that are not corrected by the last reconstruction. In particular, in Fig. S5c the CNN outputs two cells (red arrow A in Fig. S5c) whereas the first reconstruction present only one (Fig. S5b). In this case, the last reconstruction does not recover a single cell image (red arrow A in Fig. S5d) and outputs two cells instead. This last example indicates that the alternation method does not fully generalize when applied to samples of adherent cell culture.

## Discussion

In this article, we introduce a novel reconstruction approach, based on alternations between an inverse problem approach and deep learning using CNN to improve lens-free image reconstruction. We have designed the CNN to perform phase unwrapping on the acquisition of cells in suspension. CNN has been trained on synthetic datasets and we demonstrate the generalization to real samples. On a sample of PC3 cells in suspension, we show an effective phase unwrapping up to phase shift of 5$\pi$. With this method we succeeded in reconstructing cells in suspension at large cell densities of up to $\sim 1000$ cells/$\text{mm}^2$. This allows us to observe 35,000 cells simultaneously in a field of view of 30 $\text{mm}^2$ with our lens-free system. According to simulations, at this concentration, the method is quantitative for the determination of the cell refractive index (real part). Alternatively to the other CNN-based lens-free microscopy techniques^17,18^, our final image is obtained with a reconstruction step following the CNN prediction. As an advantage, this last reconstruction can correct the prediction of the CNN when it is not in agreement with the measured data. In some cases, this third step allows the reconstruction of sample different from the original training data. As an example, we have shown that our approach is successful in reconstructing the image of PC12 adherent cells. However, the alternation approach was not successful when tested on the acquisition of adherent fibroblast cells. Hence, the proposed alternation approach does not generalize to the large domain of adherent cell culture. In sum, the novel CNN-based solution allows to reconstruct the image of the cells in suspension free of phase wrapping errors. It extends thus the applicability of lens-free microscopy to the live imaging of cells in suspension.

## Method

The proposed alternation approach is a three-step algorithm (see Fig. 1). A first holographic reconstruction is initialized using a null sample, leading to the computation of a first guess of the sample image. The result of the first reconstruction is used as the input for a convolutional neural network, which has beforehand been trained to correct the object phase image from wrapping errors. The CNN is not trained with experimental image data, but instead with synthetic images. The network output is next used as initialization of the last holographic reconstruction step. In the following, we detail the different steps of the reconstruction algorithm and describe the synthetic CNN training. Next we present how we assess the reconstructions of the simulated floating cells in a quantitative manner. Finally we describe the lens-free setup and the cell lines used for the experimental validation.

### Holographic reconstruction

The holographic reconstruction generates an object description from intensity measurements. It relies on a given physical description of the object and a forward model which allows to predict the measurements for a given object (see “Physical model”). The reconstructed object is obtained with an inverse problem approach, namely a gradient-based iterative process minimizing a given criterion (see “Inverse problem approach”).

#### Physical model

**Figure 7:**
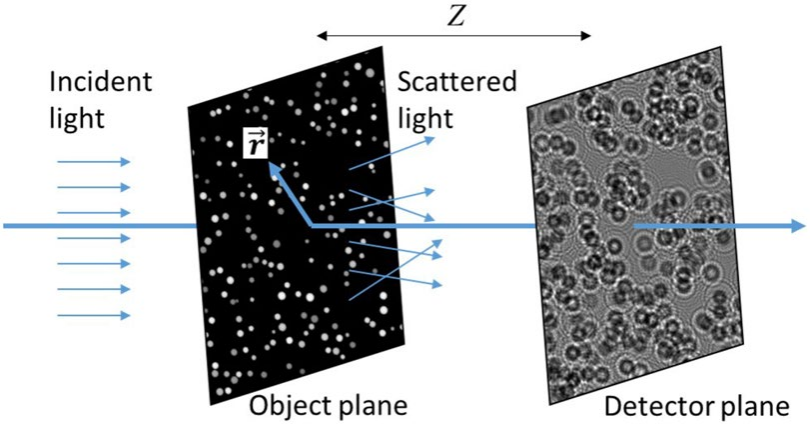
Principle of lens-free microscopy. A 2D object is illuminated by a partially coherent light. The intensity of the generated interference pattern at a distance *Z* behind the sample is recorded with a camera.

Partially coherent illumination is diffracted by the object as shown in Fig. 7. The intensity of the generated interference pattern (hologram) at a distance *Z* behind the sample is recorded with a camera. The light field, at wavelength $\lambda$, is described as a complex scalar field and the illumination light is described by a plane wave with normal incidence and with intensity normalized to one. The transmission of the sample is assumed to be directly related to *L* the optical path difference (OPD) and *A* its (negative) absorption coefficient. The light field *E* after the sample is:

1 $$\begin{aligned} E(L,A)=\exp (2i\pi L/\lambda +A) \end{aligned}$$

*i* being the imaginary unit. By using the Fresnel propagator, $h_Z(\mathbf {r})=1/(i\lambda Z)\exp (i\pi \mathbf {r}^2/(\lambda Z))$, and taking into account the partial coherence of the light source by a convolution kernel *K*, and the uniform light intensity *B* (“background”), a direct model of the intensity measurement of image *I* is set as:

2 $$\begin{aligned} I(L,A)=B\left| E(L,A) * h_Z \right| ^2*K \end{aligned}$$

where ‘*’ is the convolution operator. *I*(*L*, *A*) is the measurement used in the subsequent data processing, it is sampled by the sensor. The unknowns *L* and *A*, have the same size as *I*. There are particular difficulties related to the lens-free setup. First, for any integer *N*, $L+N\lambda$ results in the same transmission and consequently the same measurements. This will give rise to wrapping problems, as the reconstruction of *L* will be known modulo $\lambda$ values only. Second, the phase of the light field is not recorded by the detector. Therefore “half” of the information is lost during the detection process, giving rise to so-called ‘twin-image’ artefacts^4^. Furthermore, high intensity frequencies are lost due to partial coherence of the source, i.e. non-point and multi-spectral source. This is reflected in the forward model by the convolution kernel *K*. In this study, to compute such a kernel *K*, we perform two simulations on a Dirac object, one with total source coherence and one with partial source coherence (source dimension is taken into account and source spectrum is discretized, see Table 1 for source characteristics) giving rise to measurements $I_{total}$ and $I_{partial}$. *K* is extracted by deconvolving $I_{partial}$ from $I_{total}$.

#### Inverse problem approach

The inverse problem approach used to retrieve the object from intensity measurements is formulated as a regularized optimization problem. A criterion $\epsilon$ implementing a data fidelity term and a regularization term $\zeta$ for the unknowns *L* and *A* is set as:

3 $$\begin{aligned} \epsilon (L,A)=\int d\mathbf {r} \frac{\left| I(L,A)-I_{meas} \right| ^2}{I_{meas}}+\alpha \zeta (L,A) \end{aligned}$$

Where *I*(*L*, *A*) is the direct model (see Eq. 2) and $I_{meas}$ is the intensity measurement. As shown in Fig. 1, two reconstruction steps are performed in the proposed algorithm. The first reconstruction is intended to be a fast step, with kernel *K* of Eq. (2) set to a Dirac distribution, limited to a low number of iterations (20) and with no attempt to phase unwrapping. For this first reconstruction the regularisation term is:

4 $$\begin{aligned} \zeta (L,A)= \int d\mathbf {r}\left( \sqrt{\left| \frac{\partial E(L,A)}{\partial x}\right| ^2+\left| \frac{\partial E(L,A)}{\partial y}\right| ^2} + \sqrt{L^2}+10A^2(A>0)\right) \end{aligned}$$

The first term of Eq. (4) is the total variation applied to the complex light field *E* to favor sharp edges. The spatial derivative of this term is not sensitive to $\lambda$ jumps of *L* and will not propagate wrapping artefacts. The second term is a sparsity constraint on *L*. The third term limits the absorbance of the object to positive values. In comparison with the first reconstruction, the second reconstruction performs a larger amount of iterations (70) and takes into account the coherence of the illumination source, using a kernel *K*. In addition, the regularization term is more complex. This is the optimization scheme that has been previously published in^20^. The regularization term in the second reconstruction is set to:

5 $$\begin{aligned} \begin{aligned} \zeta (L,A)=&\int d\mathbf {r}\left( \sqrt{\left( \frac{\partial L}{\partial x}\right) ^2 + \left( \frac{\partial L}{\partial y}\right) ^2} \right. \\&+ \left. \frac{1}{5}\sqrt{L^2}+\frac{1}{5}(\Delta A)^2+10A^2(A>0)+L^2(L<0)\right) \end{aligned} \end{aligned}$$

Compared to Eq. (4), the first term is now expressed with variable *L*, the OPD. This is possible since the object is assumed to have been unwrapped by the performed CNN step. The third term favors a smooth map of *A* and the last term implements the constraint that the unwrapped OPD must be positive. As shown previously^7^, the gradient of $\epsilon (L,A)$ can be analytically computed. The minimization of the criterion is performed by a conjugate gradient optimizer.

### CNN for phase unwrapping of lens-free acquisition

The CNN step (see Fig. 1) takes place between the two holographic reconstruction steps and is processing the results of the first reconstruction to correct phase wrapping errors. Indeed, data fidelity term being insensitive to phase wrapping error, a regularized criterion such as defined in Eq. (3) will present local minima, which cannot be handled by gradient-based optimization. To overcome these local minima, we have trained a CNN to perform specifically phase unwrapping. The CNN needs to be trained on a set of input/target object pairs with phase wrapping errors. To this aim, we generated synthetic images of cells in suspension and the corresponding first reconstructions.

#### Synthetic data

**Figure 8:**
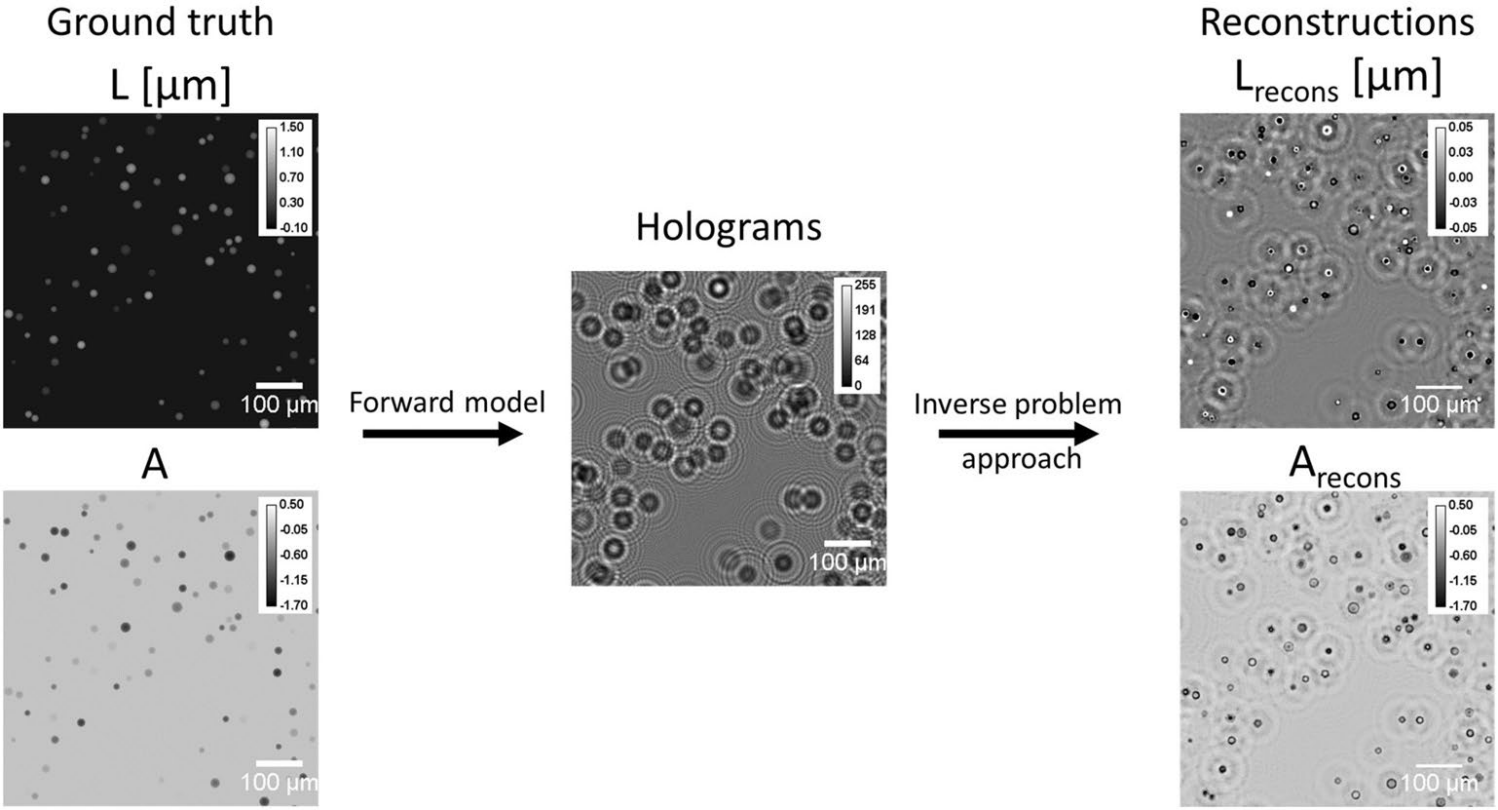
Overview of the synthetic data generation method. A pair of synthetic images (ground truth) is generated, representing cells in suspension (*L* denotes the optical path difference and *A* the absorption). Using Eq. (2), the simulated intensity measurement image *I* is obtained for a given sample-to-sensor distance *Z*. The first reconstruction applied to *I* generates the simulated images $L_{recons}$ and $A_{recons}$. The presented images are $400\times 400$ pixels ($1.67\,\upmu\text{m}$ pitch) crops of an image with $10^3$ cells corresponding to a density of 358 cells/$\text{mm}^2$.

We generated synthetic images representing cells in suspension, with homogeneous spheres of random radius and with complex refractive index (see Fig. 8 and see Table 1). The relationship between OPD *L*, the absorption coefficient *A* and the refractive index of the cell *n*, the refractive index of the surrounding medium $n_{medium}$ and the thickness of the cell *T* is given by:

6 $$\begin{aligned} \begin{aligned} L(x,y)&=T(x,y)Re(n-n_{medium})=T(x,y)\Delta n_r\\ A(x,y)&=-2\pi \frac{T(x,y)}{\lambda }Im(n-n_{medium})=-2\pi \frac{T(x,y)}{\lambda }\Delta n_i \end{aligned} \end{aligned}$$

Based on Eq. (2), the intensity measurement images *I* in the sensor plane are simulated for a given sample-to-sensor distance *Z*, coherence of the illumination source and background *B*. Furthermore, to mimic the noise in the acquisition process, Poisson noise is added to the simulated hologram *I*. The number of cells is chosen randomly between 500 and 5000 (corresponding to a density range of 179–1792 cells/mm^2^). Their radii are uniformly drawn between 5 to $20 \,\upmu\text{m}$, real and imaginary parts of the refractive index are drawn from a uniform distribution between 0.01 to 0.05 and 0 to 0.005 respectively. Figure 8 shows example images of the synthetic data. The results of the holographic reconstruction differ notably from the ground truth images, due to wrapping errors and incomplete physical modelling (kernel *K* is set to Dirac in the forward model). This result is foreseeable and not a problem *per se*. The reconstruction merely aims at producing a valid object to be used as an input for the CNN network.

**Table 1:** List of setup and simulation parameters used to generate the synthetic dataset.

Setup parameter	Values	Sim. param.	Values
Sample-source dist.	$50\,\text{mm}$	Image size	1000 × 1000
Sample-sensor dist. *Z*	1270 $\upmu\text{m}\pm 5\%$	Cells number	500–5000
Source wavelength $\lambda$	$0.450\pm 0.015\,\upmu\text{m}$	Radius	5–20 μm
Source diameter	50 $\upmu\text{m}$	$\Delta n_r$	0.01–0.05
Sensor pixel pitch	1.67 $\upmu\text{m}$	$\Delta n_i$	0–0.005
Convolution Kernel *K*	1.75 $\upmu\text{m}$	Backg. *B*	105 $\pm \, 30\%$

*Sim. param.* simulation parameter, *dist.* distance, *Backg.* background in grey levels.

#### Training of CNN

**Figure 9:**
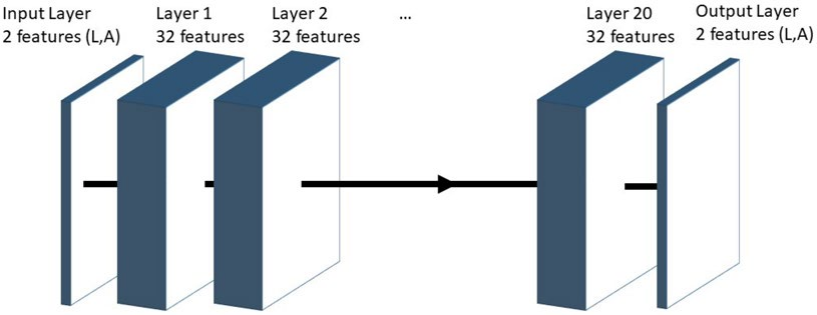
Design of the 20 layers convolutional neural network used for unwrapping of the holographic reconstruction. Each layer consists of three sub-layers, $(5\times 5)$ convolution layers, a batch normalization layer and a ReLU activation layer. No dimension changes are performed inside the network.

We constructed a simple CNN network consisting of 20 repetitions of a three layers pattern, namely a convolution layer ($5 \times 5$ pixel, 32 features), a batch normalization layer and a ReLU activation function (see a sketch of the CNN structure in Fig. 9). The final layer before the regression layer was a convolution layer with 2 features (*L* and *A* outputs) so as to match the ground truth image dimensions. The network does not perform any dimension changes, allowing images of any size to be used as training input. To train a CNN for unwrapping lens-free reconstructed phase images, the ground truth consisted of a synthetic dataset of 1000 image pairs ($1000\times 1000$ pixels for *L* and *A*) of cells in suspension. The CNN input contains the corresponding reconstructed images ($L_{recons}$ and $A_{recons}$). Two sets of vignettes (size $121\times 121$) were extracted randomly from the reconstructed images and used during the CNN training. CNN training was implemented with the Matlab deep learning library using the Adam optimizer. We used 12,800 sets of vignettes per epoch (a set being a vignette couple $(L_{recons},A_{recons})$ as input and a corresponding vignette couple as ground truth). Learning rate was set to 0.0001 and training was conducted over 10 epochs, lasting 10 h on a PC workstation with a single NVIDIA GTX Titan GPU.

### Evaluation of the results obtained on synthetic datasets

The simulated reconstructions have been quantitatively compared to the known ground truth, considering the cell relative refractive index values. From the reconstruction $(L_{recons},A_{recons})$, it is possible to retrieve for each cell the real imaginary parts of the relative refractive index according to the formula:

7 $$\begin{aligned} \begin{aligned} \Delta n^{(k)}_{r\_recons} & = \frac{1}{V^{(k)}} \int _{S^{(k)}}d\mathbf {r}.L_{recons} \\ \Delta n^{(k)}_{i\_recons}&=-\frac{\lambda }{2\pi V^{(k)}}\int _{S^{(k)}}d\mathbf {r}.A_{recons} \end{aligned} \end{aligned}$$

where *k* is the index of the cell, $S^{(k)}$ the integration domain calculated knowing the position and radius of the cell, and $V^{(k)}$ the cell volume.

### Description of the lens-free setup

To evaluate the alternation reconstruction method on experimental data, we carried out measurements using a Cytonote lens-free setup (Iprasense). Illumination is provided by a monochromatic LED source (at wavelength $\lambda =457\,\text{nm}$ with a spectral width of $20\,\text{nm}$) located $50\,\text{mm}$ away from the sample. A CMOS detector of $6.4\times 4.6\,\text{mm}^2$ with $3840 \times 2748$ pixels ($1.67\,\upmu\text{m}$ pitch) was used to measure the diffraction patterns at a distance $Z=1{-}4\, \text{mm}$ from the sample.

### Description of the cell samples

PC3 cells were cultured in RPMi 1640 medium, containing 10% fetal bovine serum, 50 ng/mL geneticine and 1% of PenStrep. Cells were passed twice a week, using a 1:6 dilution. Cell density for experiments was typically $1 \times 10^5$ cells/mL. PC3 cells contained a GFP expressing vector. PC12 cells were cultured in proliferation medium, consisting of RPMi 1640 medium, supplemented with 10% Hi Horse Serum, 5% fetal bovine serum and 1% of PenStrep. They have been passed twice a week at a 1:10 dilution. To perform differentiation into a neuronal phenotype, cells were plated at a density of $1 \times 10^4$ cells/mL in differentiation media, consisting of RPMi medium supplemented with 1% Hi Horse Serum and 50 ng/mL NGF.

## Supplementary information

Supplementary Information.
