# Supplementary material for: Alternation of inverse problem approach and deep learning for lens-free microscopy image reconstruction
Source: Sci Rep. 2020 Nov 19;10:20207. doi: 10.1038/s41598-020-76411-9 (PMC7678858; doi:10.1038/s41598-020-76411-9)
Supplement: Supplementary file 1 — Supplementary Information. [file 41598_2020_76411_MOESM1_ESM.docx]

**Alternation of inverse problem approach and deep learning for lens-free microscopy image reconstruction**

Hervé L.^1^, Kraemer D. C.^1^, Cioni O.^1^, Mandula O.^1^, Menneteau M.^1^, Morales, S.^1^, and Allier C.^1^,

^1^ Univ. Grenoble Alpes, CEA, LETI, DTBS, F-38000 Grenoble, France

^*^cedric.allier@cea.fr

**Supplementary informations**

**
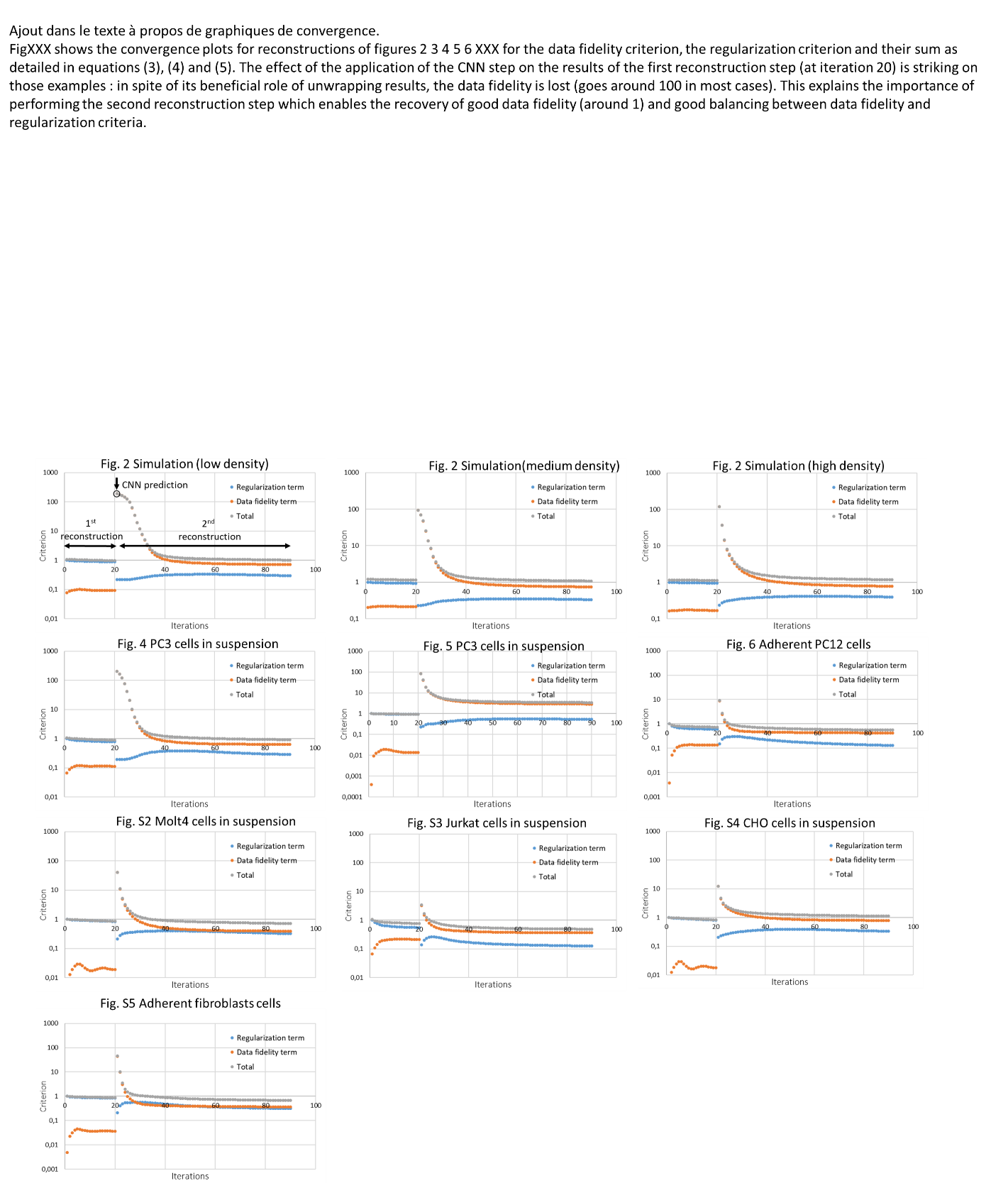
**

Figure S1 : Convergence plots of the reconstruction as a function of the iteration for different cases depicted in respectively figures 2, 3, 5, and 6 of the manuscript and figures S2, S3, S4 and S5 of the supplementary informations. The orange, blue and grey curves respectively represent the data fidelity criterion, the regularization criterion and the total of the two. These criteria are detailed in the manuscript under equations (3), (4) and (5).  As depicted in the top left figure, the first 20 iterations are related to the first reconstruction step and the next 70 iterations are related to the second reconstruction step. In between the CNN step occurs after iteration 20.


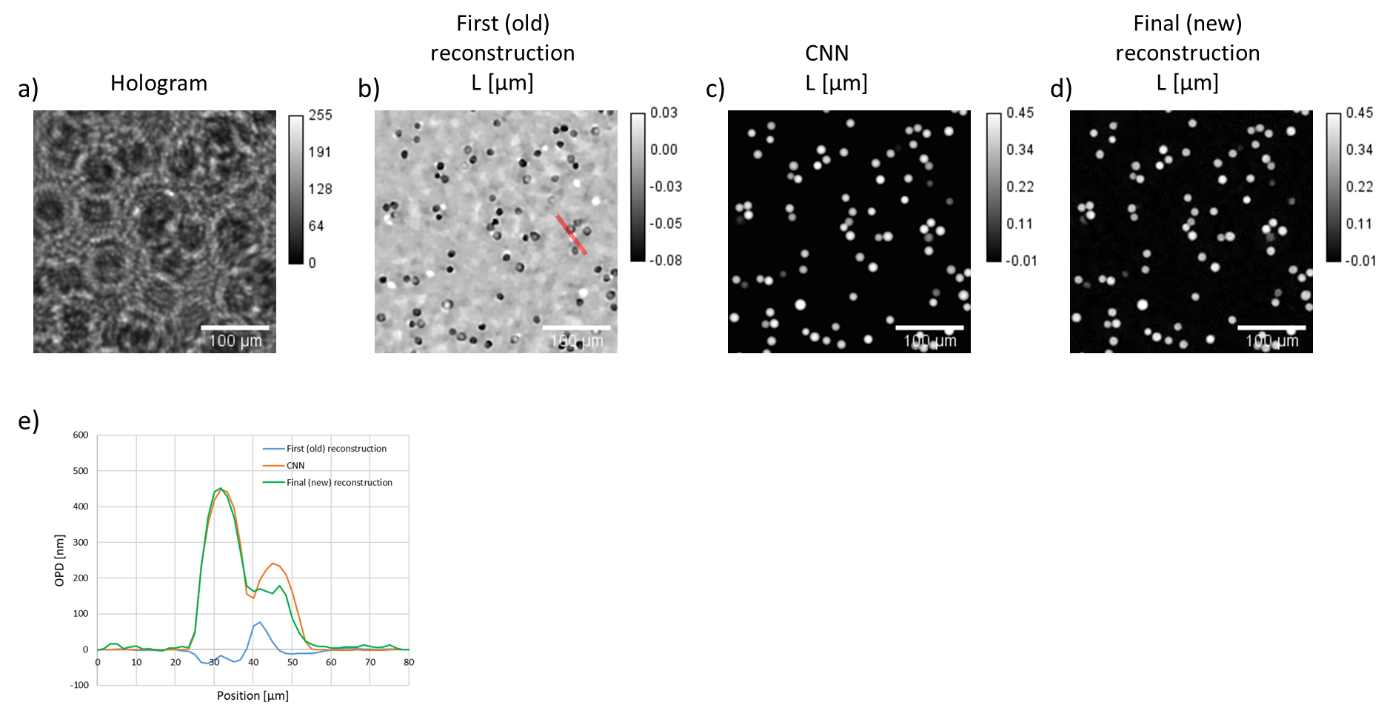
Fig. S2 MOLT4 cells in suspension. Reconstruction of a cropped image (350µmx350µm) taken out of the full field of view (6410µmx4590µm) (a) Raw acquisition. (b) First (old) reconstruction result. (c) CNN output result. (d) Final (new) reconstruction result. (e) OPD profile through two cells (red line 1 in b).


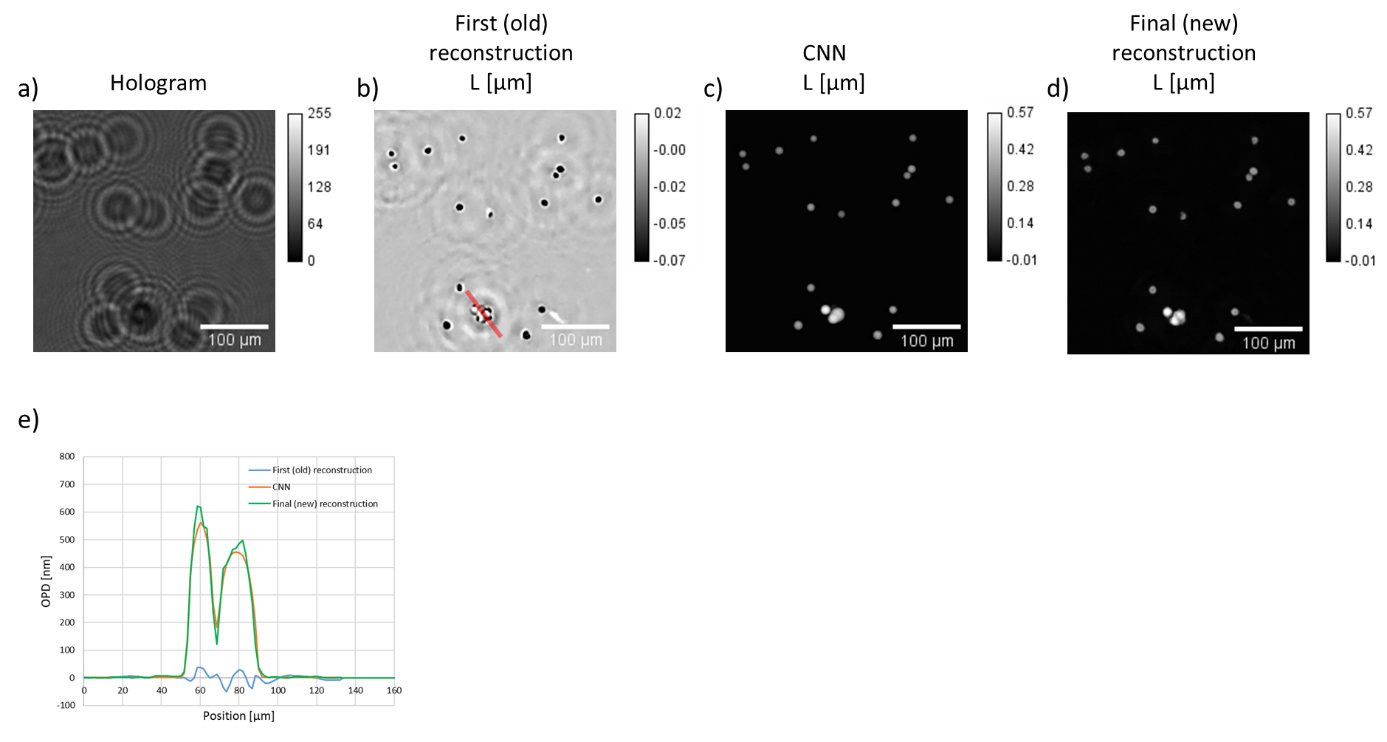
Fig. S3 Jurkat cells in suspension. Reconstruction of a cropped image (350µmx350µm) taken out of the full field of view (6410µmx4590µm) (a) Raw acquisition. (b) First (old) reconstruction result. (c) CNN output result. (d) Final (new) reconstruction result. (e) OPD profile through two cells (red line 1 in b).


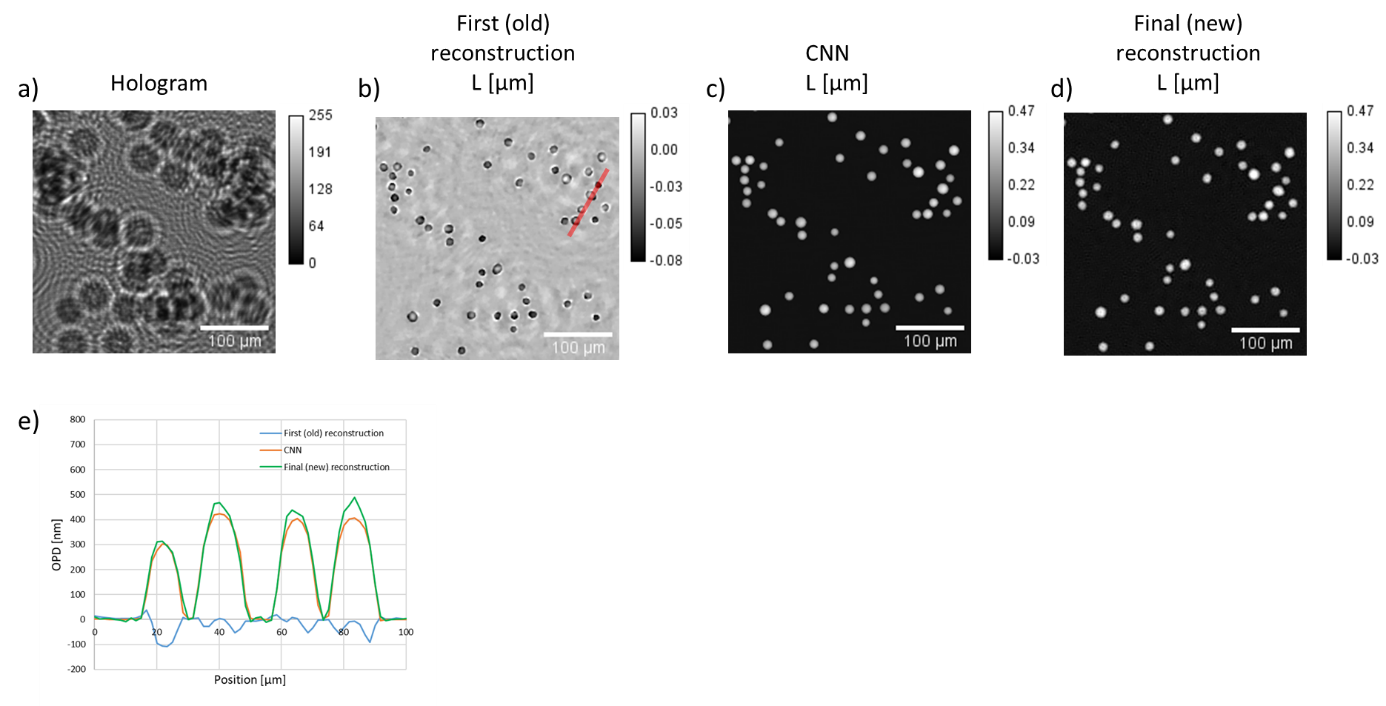


Fig. S4 CHO cells in suspension. Reconstruction of a cropped image (350µmx350µm) taken out of the full field of view (6410µmx4590µm) (a) Raw acquisition. (b) First (old) reconstruction result. (c) CNN output result. (d) Final (new) reconstruction result. (e) OPD profile through four cells (red line 1 in b).


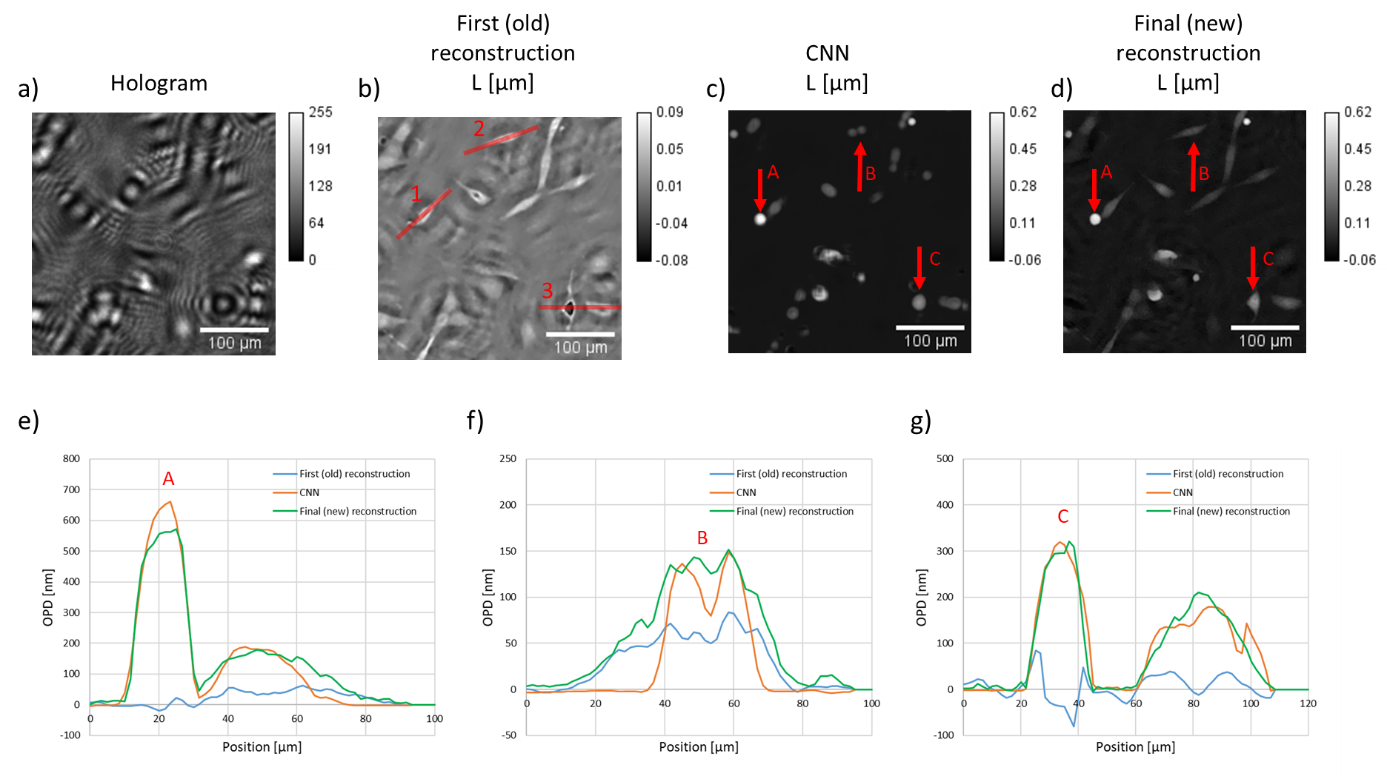


Fig. S5 Culture of adherent Fibroblasts cells. Reconstruction of a cropped image (350µmx350µm) taken out of the full field of view (6410µmx4590µm) (a) Raw acquisition. (b) First (old) reconstruction result. (c) CNN output result. (d) Final (new) reconstruction result.

(e) OPD profile through one cell (red line 1 in b). Along this profile, the CNN output (c) is wrong. It produces two cells instead of one as one can deduct from the first reconstruction (b). The arrow A in (c) points one the cell ‘hallucinated’ by the CNN, a rounded cell with large OPD value (up to 600). The last reconstruction (d) does not correct this error introduced by the CNN. Especially it does not remove the rounded cell with large OPD values ‘hallucinated’ by the CNN output.

(f) OPD profile through one cell (red line 2 in b). Along this profile, the CNN output (c) is wrong. It produces two cells instead of one as one can deduct from the first reconstruction (b). The arrow B in (c) points these cells predicted by the CNN. The last reconstruction (d) does correct the error introduced by the CNN. The final reconstruction presents one cell instead of two in the CNN output (c). Phase unwrapping is correctly performed in the cell body area.

(g) OPD profile through two cells (red line 3 in b). The CNN output (see arrow C in (c)) does not reflect the irregular shape of the cells as shown in the first reconstruction. The last reconstruction (d) does correct the error introduced by the CNN. The final reconstruction presents cells with irregular shape more in line with the first reconstruction (b). Phase unwrapping is correctly performed in the body area of the two cells.
